# Crosstalk Between *BmToll9-2*, the Toll Pathway, and Antimicrobial Peptides in the Silkworm (*Bombyx mori*) Larval Fat Body

**DOI:** 10.3390/insects17090905

**Published:** 2026-08-28

**Authors:** Ruixuan Lin, Shiyuan Li, Hui Lv, Qiuying He, Xintong Wu, Ruiling Wu, Qingrong Li, Jisheng Liu

**Affiliations:** 1 School of Life Sciences, Guangzhou University, Guangzhou 510006, China; 2 The Sericultural and Agri-Food Research Institute of the Guangdong Academy of Agricultural Sciences, Guangzhou 510610, China

**Keywords:** *Bombyx mori*, *BmToll9-2*, RNAi, Toll pathway, antimicrobial peptides, immune response

## Abstract

Insects lack adaptive immunity and rely exclusively on innate immune system for pathogen defense. However, the specific role of *BmToll9-2*, a key Toll receptor in the silkworm (*Bombyx mori*), in mediating immune responses, particularly against bacterial challenges in the larval fat body, remains incompletely understood. To address this gap, this study employed a combination of molecular approaches, including RNA interference (RNAi) targeting *BmToll9-2*, bacterial challenges with heat-inactivated *Escherichia coli* (Gram-negative) and *Staphylococcus aureus* (Gram-positive), and quantitative real-time PCR (qPCR) to assess the transcriptional changes of *BmToll9-2* and immune-related genes in the fat body, a key immune tissue. Quantitative analysis showed that after *BmToll9-2* RNAi, bacterial challenges significantly upregulated *BmToll9-2* expression in the fat body at 12 h post-challenge, whereas *BmToll9-2* silencing alone notably downregulated most downstream signaling genes of the Toll pathway by 53.07–83.14%, as well as 11 immune effector genes, including antimicrobial peptide genes, by 64.48–93.40%. Importantly, feeding bacteria post-RNAi reversed these downregulations: *E. coli* induced a stronger upregulation of both signaling and effector genes compared to *S. aureus*. This study provides transcriptional evidence that *BmToll9-2* may act as a positive regulator of the Toll pathway signaling and antimicrobial peptides in silkworm larval fat body, facilitating robust and rapid immune signaling. This study deepens our understanding of Lepidopteran innate immunity by elucidating key molecular mechanisms, thereby providing a solid foundation for refining RNAi-based pest control strategies.

## Simple Summary

Silkworms rely on innate immunity to resist pathogens, with Toll receptors undertaking core immune regulatory roles. Previous studies have explored several silkworm Toll genes, yet how *BmToll9-2* mediates immune responses in larval fat body is still unknown. We knocked down *BmToll9-2* via RNA interference and stimulated silkworms with two typical bacterial species. The suppression of *BmToll9-2* restrained the activation of immune signaling and antimicrobial peptide production. Subsequent bacterial treatment upregulated the expression of immune genes, and distinct induction strengths were observed between different bacteria. This study provides transcriptional evidence that *BmToll9-2* may act as a positive regulator of fat body immunity through the Toll cascade. These outcomes enrich our understanding of Lepidoptera Toll immune mechanisms and lay a theoretical basis for developing RNAi pest management techniques.

## 1. Introduction

As one of the most taxonomically diverse groups of organisms on Earth, insects exhibit remarkable adaptability to diverse environments and exceptional competence in resisting pathogen invasion, primarily attributed to their highly evolved innate immune system. This system comprises two synergistic components: cellular immunity and humoral immunity, which counteract various pathogens challenges [1]. Cellular immunity eliminates pathogens primarily through hemocyte-mediated processes, including phagocytosis, nodulation, encapsulation, and coagulation [2]. Humoral immunity functions mainly via antimicrobial molecules and signaling pathways in the hemolymph. The Toll, immune deficiency (IMD), and Janus kinase/signal transducer and activator of transcription (JAK/STAT) pathways are the three most extensively studied [3] and are closely associated with coagulation, melanization, and reactive oxygen species production [4].

Upon pathogen invasion, pattern recognition receptors (PRRs) in the hemolymph recognize pathogen-associated molecular patterns (PAMPs) [5]. This recognition triggers a complex serine protease cascade, culminating in the cleavage of a cytokine precursor Spätzle and the release of its active ligand. The activated Spätzle ligand binds to Toll receptors on the cell membrane, initiating the Toll pathway. The Toll pathway represents an ancient and highly conserved signaling cascade in insect innate immunity, primarily responsible for defending against fungal and Gram-positive bacterial infections [6]. These components form a signaling complex that activates NF-κB-like transcription factors, which translocate to the nucleus, bind target gene promoters, and induce the expression of antimicrobial peptide (AMP) genes. AMPs such as Defensin and Drosomycin are secreted into the hemolymph, where they act directly on pathogens, often by disrupting pathogen cell membranes, to mediate elimination [7].

Toll receptors, a subset of PRRs, are characterized by an extracellular leucine-rich repeats (LRRs) array and an intracellular Toll-interleukin-1 receptor (TIR) domain [7]. The first Toll receptor, discovered during studies of the embryonic development of *Drosophila melanogaster*, was shown to play a central role in establishing embryonic dorsoventral polarity [8]. To date, nine genes encoding Toll-related receptors have been identified in the *Drosophila* genome [9], collectively constituting a robust innate immune defense framework [10]. Nevertheless, a “Diptera bias” has long dominated the understanding of Toll cascades, whereby our knowledge relies excessively on the fruit fly model while overlooking the functional diversity across other insect orders [11]. Within Lepidoptera, dramatic gene expansion events have occurred in the Toll receptor family. For instance, *Bombyx mori* Toll9 has been characterized as a PRR capable of directly detecting PAMPs, a feature analogous to mammalian Toll-like receptor 4 (TLR4). This finding overturns the conventional paradigm that insect Toll receptors are only indirectly activated via the Spätzle ligand [12]. Structural characterization of silkworm Toll9 reveals its capacity to directly bind microbial moieties, implying that certain Lepidopteran Toll receptors may retain ancestral pathogen-sensing functions or acquire supplementary recognition capacities through neofunctionalization during early Lepidopteran evolution [12,13]. Moreover, the diversification of Toll pathway components is also reflected in downstream signaling molecules across Lepidoptera. In *Lymantria dispar*, 11 Toll-like receptor genes and five downstream cascade components have been identified, which exhibit distinct transcriptional induction profiles upon immune challenge, indicative of intricate regulatory networks [14]. Research focusing on the Toll superfamily of the Lepidopteran model organism *Bombyx mori* has advanced progressively. The *B. mori* genome encodes 14 Toll family members [15]. Phylogenetic analysis indicates that six BmTolls belong to the immune-related clade, while others are involved in diverse biological processes [16].

The silkworm serves as both a critical economic insect and a model organism for investigating innate immune mechanisms [17]. Among the BmToll members, functional studies of the *BmToll9* subfamily (comprising *BmToll9-1* and *BmToll9-2*) are particularly comprehensive. Previous studies have shown that *BmToll9-1* is expressed across all developmental stages, with high expression in the midgut, suggesting its potential involvement in local intestinal immunity. Its expression is significantly induced by challenges with *Escherichia coli* and *Beauveria bassiana* [18]. *BmToll9-1* can also act as a PRR, recognizing lipopolysaccharides (LPS) through interactions with *BmMD-2A* and *BmMD-2B*, to activate immune-related gene expression [12]. Our team has reported that *BmToll9-1* is highly expressed in the midgut. While injection of double-stranded RNA (dsRNA) and LPS inhibits its expression, oral administration of dsRNA has no significant effect due to degradation by midgut fluid. Additionally, dsRNA injection also induces the expression of RNA interference (RNAi)-related genes *Dcr2* and *Ago2* [19]. Subsequent studies further demonstrated that *BmToll9-1* regulates the transcriptional response of Bm5 cells to dsRNA and LPS, promoting expression of the RNAi pathway gene Dicer2, while simultaneously suppressing the transcriptional induction of IMD and JAK-STAT pathway genes as well as AMP genes, without directly participating in the RNAi [20]. Furthermore, *BmToll9-1* functions as a positive immune regulator. Its silencing leads to reduced larval and cocoon size and weight, downregulates the expression of Toll pathway signaling genes and effector genes, and diminishes hemolymph antibacterial activity against *E. coli* [21]. Notably, following RNAi-mediated silencing, *E. coli* can reactivate *BmToll9-1*, thereby restoring larval growth and upregulating the expression of downstream Toll pathway signaling genes and immune effector genes [22]. In terms of *BmToll9-2*, its structure includes a signal peptide and LRR domains, and its expression is induced by *E. coli*, LPS, *Staphylococcus aureus* and peptidoglycan (PGN) [16]. Silencing *BmToll9-2* inhibits silkworm growth, while bacterial challenges can upregulate its expression and rescue this growth defect [16]. We also found that, similar to *BmToll9-1*, *BmToll9-2* serves as a positive regulator of the humoral immune response. Its silencing reduces the expression of Toll pathway genes and antibacterial activity, whereas bacterial challenges induce its expression, activate downstream immune genes, and elicit a preferential response to *E. coli* [23]. Meanwhile, *BmToll9-2* is transcriptionally upregulated in the midgut, fat body and epidermis by exogenous non-specific dsRNA [24].

Evidence indicates that immune responses in insects exhibit remarkable tissue specificity. In Lepidoptera, the midgut and fat body represent two functionally distinct immune compartments. The midgut, as the frontline barrier directly exposed to the external environment and gut microbiota, mounts a localized and tolerance-oriented immune response [25]. Studies have demonstrated that AMP production within the gut is tightly constrained by negative regulatory factors, such as the Caudal homeobox transcription factor, to prevent excessive collateral damage to commensal microbiota, thereby preserving intestinal homeostasis [26]. Diverging from the localized defense mounted by the intestine, the fat body acts as the primary executor of systemic humoral immune reactions in lepidopteran insects [27]. Upon pathogen invasion breaching the intestinal epithelial barrier and entering the hemocoel, fat body cells are activated by circulating humoral signals, such as the Spätzle cytokine. This activation triggers the massive synthesis and secretion of AMPs into the hemolymph, thereby orchestrating a systemic bactericidal effect [28].

While our previous studies focused on the midgut [23], given the fundamental differences between local gut immunity and systemic fat body immunity, in terms of activation signals, regulatory mechanisms, and physiological outcomes, the function of *BmToll9-2* in the fat body cannot be simply extrapolated from midgut data. Whether *BmToll9-2* acts through the same or distinct molecular mechanisms in these two tissues, and whether its regulatory strength differ between local and systemic immune compartments, are questions that remain unanswered. Therefore, this study investigates the function of *BmToll9-2* within the larval fat body. By employing RNAi to silence *BmToll9-2*, combined with bacterial challenges using both Gram-negative bacterium *E. coli* and Gram-positive bacterium *S. aureus*, and utilizing quantitative real-time PCR (qPCR) to monitor transcriptional changes in key Toll pathway signaling genes and effector genes, this research aims to elucidate whether and how *BmToll9-2* acts as a regulator of the Toll pathway and downstream immune effectors in the fat body. The findings will clarify its regulatory role in the systemic immune defense of Lepidopteran insects, particularly in fat body-mediated humoral immunity, thereby deepening the understanding of insect innate immunity and providing a novel theoretical foundation for RNAi-based pest management strategies.

## 2. Materials and Methods

### 2.1. Insect Rearing

The silkworms (P50 strain) employed in the study were supplied by the Guangdong Academy of Agricultural Sciences. Larvae were hatched and reared on fresh mulberry leaves under controlled conditions: relative humidity at 75 ± 5%, temperature at 25 ± 1 °C, and a photoperiod of 12 L:12 D [23].

### 2.2. Synthesis of dsRNA and RNAi Protocol

T7 promoter sequences were introduced into fragments of green fluorescent protein (GFP) and *BmToll9-2* (Table 1). Following amplification, dsRNA was synthesized using the T7 RiboMAX Express RNAi system (Promega, Madison, WI, USA), yielding dsRNA of GFP and *BmToll9-2*, followed by dilution of both dsRNA solutions to a concentration of 500 ng/μL. Ds*BmToll9-2* was designated as the experimental group, while dsGFP served as the control group [29]. On day 1 of the 5th instar, 10 μL of ds*BmToll9-2* (a total amount of 5 μg) was injected between the 2nd and 3rd abdominal segments as our recent report (*n* = 50 per treatment for each repeat) [16,23]. The same injection protocol was adopted for the control group. The experiment included three independent replicates, with 50 larvae per replicate. Fat body samples were collected at 3 and 12 h post-injection for subsequent analysis.

### 2.3. Bacteria Culturing and Bacterial Challenge

Gram-negative bacterium *E. coli* and Gram-positive bacterium *S. aureus* strains from our laboratory were inoculated into Luria-Bertani (LB) broth and incubated at 37 °C for 2–3 h until the absorbance at optical density of 600 (OD_600_) value reached 0.5. Bacterial cultures were centrifuged at 8000× *g* for 5 min. The collected cell pellets were resuspended in sterile water (~5 × 10^8^ cells/mL) and heat-inactivated at 80 °C for 5 min. Following RNAi treatment, each larva was provided with a mulberry leaf disk of equal size coated with 10 μL of the bacterial suspension (*n* = 50 per treatment for each repeat) [23].

### 2.4. RNA Extraction and cDNA Synthesis

Total RNA was extracted from fat body tissue samples after RNAi and bacterial treatment above using RNAiso Plus kit (TaKaRa, Kusatsu, Japan). Extracted RNA concentration and purity were accessed via NanoDrop One (Thermo Fisher, Wilmington, DE, USA). To eliminate genomic DNA contamination, the quantified RNA was digested with RNase-free DNase I (TaKaRa). Following the PrimeScript RT Reagent Kit (TaKaRa) instructions, treated RNA served as template for reverse transcription to synthesize first-strand cDNA [23].

### 2.5. Quantitative Real-Time PCR (qPCR)

qPCR was performed using GoTaq qPCR Master Mix (Promega, Madison, WI, USA) on a CFX Connect Real-Time System (Bio-Rad, Hercules, CA, USA). The thermal cycling protocol was as follows: 95 °C for 2 min, followed by 40 cycles of 95 °C for 15 s and 57 °C for 30 s. Primers for Toll pathway signaling genes and effector genes are listed in Table 1. Translation initiation factor 4A (*BmTIF4A*) and translation initiation factor 3 subunit 4 (*BmTIF3s4*) served as reference genes [30]. Transcript levels were normalized via geometric averaging of reference gene expressions and calculated using the 2^−ΔΔCT^ method [31]. Fold induction was calculated as the ratio of expression in the ds*BmToll9-2* treatment group to that in the dsGFP control group (treatment/control).

### 2.6. Data Analysis

Statistical analysis was conducted using SPSS 26.0. Prior to two-way analysis of variance (ANOVA), the assumptions of normality and homogeneity of variances were assessed using the Shapiro-Wilk test and Levene’s test, respectively. The two-way ANOVA was performed with “RNAi treatment” (dsGFP vs. ds*BmToll9-2*) as fixed factors, and their interaction was tested. Post-hoc comparisons were performed using Tukey’s HSD test for multiple comparisons. Data visualization was performed with GraphPad Prism 10.0. All data are presented as mean ± standard deviation (SD) from three biological replicates.

## 3. Results

### 3.1. Bacterial Challenges Following RNAi Induced the Transcription of BmToll9-2

**Figure 1:**
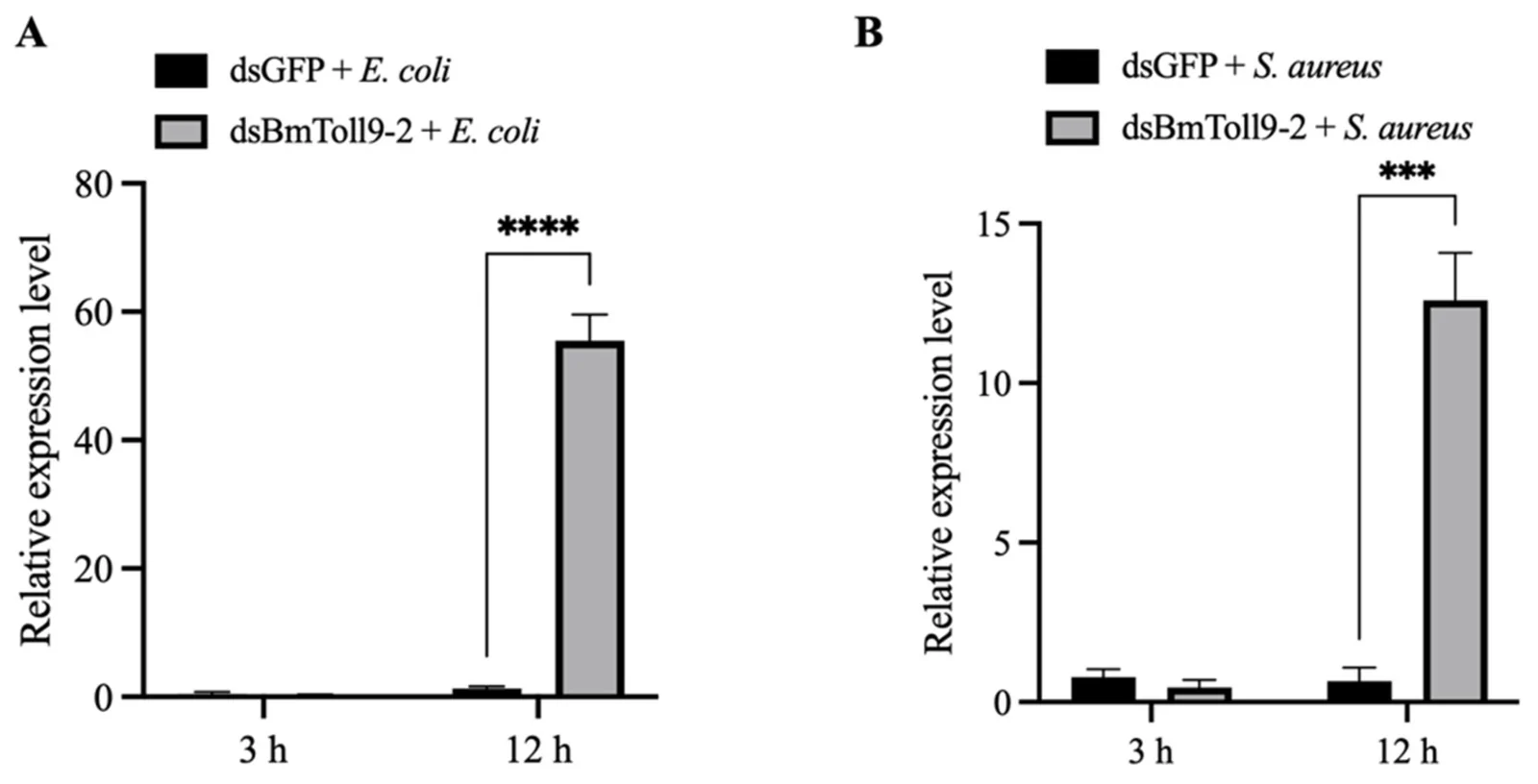
The relative expression of *BmToll9-2* following challenges with heat-treated (**A**) *E. coli* or (**B**) *S. aureus* after the RNAi of *BmToll9-2* in the fat body. Ds*BmToll9-2* was injected to the 5th-instar larvae, and dsGFP served as the control. The relative expression of *BmToll9-2* was detected at 3 and 12 h after dsRNA injection and compared with that of the control. Data are represented as means ± standard deviations. Asterisks indicate significant differences in dsGFP injection groups (two-way ANOVA followed by Tukey’s HSD post-hoc test): *** *p* < 0.001; and **** *p* < 0.0001.

To assess the expression level of *BmToll9-2* after bacterial challenges, silkworm larvae injected with ds*BmToll9-2* were fed with heat-inactivated *E. coli* and *S. aureus*, respectively, while silkworm larvae injected with dsGFP following oral bacteria administration served as controls. The relative expression levels of *BmToll9-2* were examined at 3 h and 12 h post-feeding (Figure 1).

Injection of ds*BmToll9-2* silenced the expression of *BmToll9-2* in fat body in our previous report [16]. Challenged with *E. coli* at 3 h after silencing of *BmToll9-2* did not alter the expression in both groups. However, challenged with *E. coli* at 12 h induced the expression of *BmToll9-2* at 43.21-fold at the RNAi group compared to the control group (Figure 1A).

Similarly, challenged with *S. aureus* at 3 h after silencing of *BmToll9-2* didn’t alter the expression in both groups either. However, challenged with *S. aureus* at 12 h induced the expression of *BmToll9-2* at 19.08-fold at the RNAi group compared to the control group (Figure 1B). When comparing the two bacteria, the expression of *BmToll9-2* induced by *E. coli* was 4.41-fold higher than that induced by *S. aureus* in the RNAi group.

### 3.2. Silencing of BmToll9-2 Downregulated the Toll Pathway Signaling Genes

**Figure 2:**
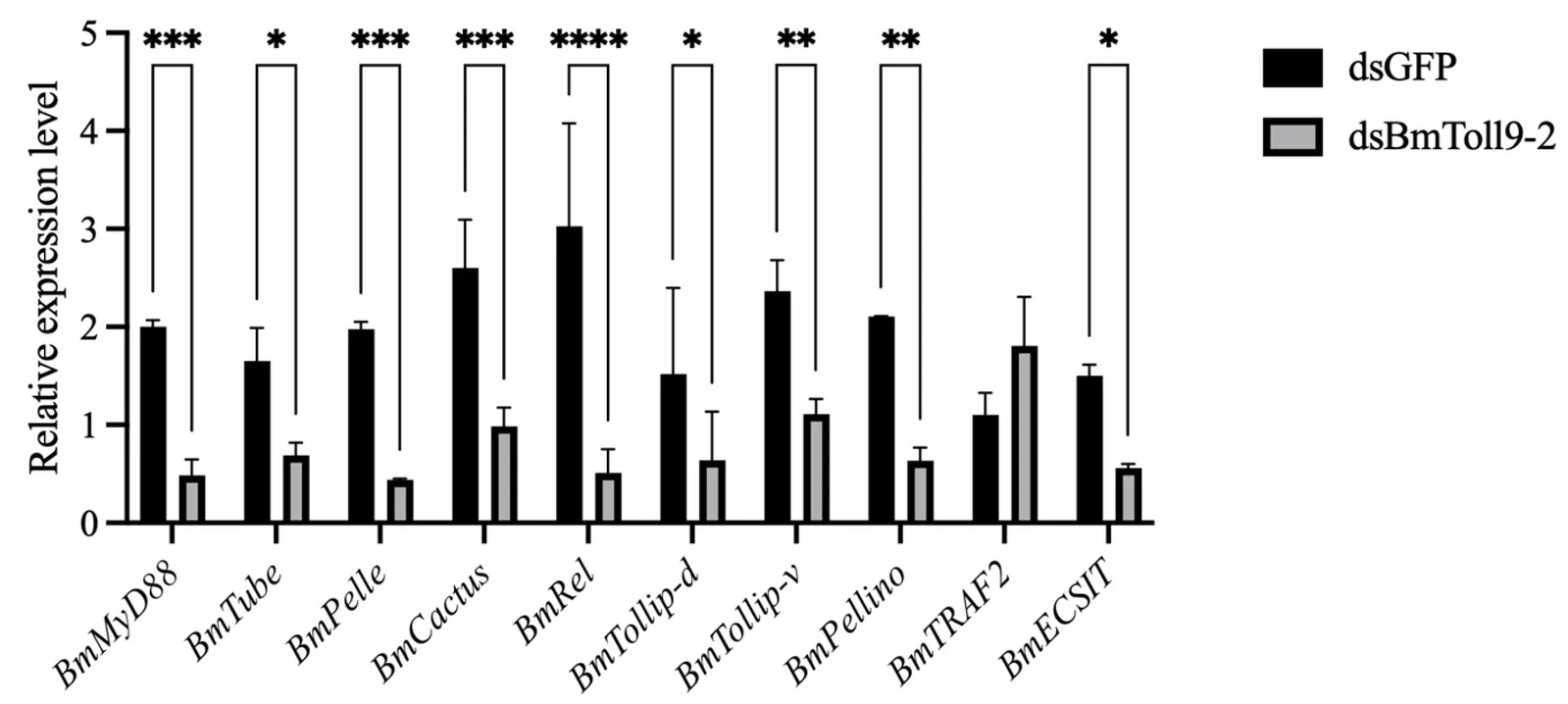
The relative expression of signaling genes in the Toll pathway after the RNAi of *BmToll9-2* in the fat body. 5th-instar larvae were injected with ds*BmToll9-2*, and dsGFP served as a control. Data are represented as means ± standard deviations. Asterisks indicate significant differences from dsGFP injection groups (two-way ANOVA followed by Tukey’s HSD post-hoc test): * *p* < 0.05; ** *p* < 0.01; *** *p* < 0.001; and **** *p* < 0.0001.

In our previous report, injection of ds*BmToll9-2* effectively downregulated the expression of the Toll pathway signaling genes in the midgut [23]. To clarify the regulatory role of *BmToll9-2* in the downstream Toll pathway of silkworm larval fat body, the expression level changes of key downstream signaling genes were assessed post-RNAi (Figure 2).

Compared to the dsGFP control group, ds*BmToll9-2* treatment significantly decreased the expression levels of most Toll pathway signaling genes, except *BmTRAF2*. Specifically, *BmMyD88*, *BmTube*, *BmPelle*, *BmCactus* and *BmRel* were reduced by 75.75%, 58.18%, 77.72%, 62.12% and 83.14%, respectively; *BmTollip-d*, *BmTollip-v*, *BmPellino* and *BmECSIT* were decreased by 57.89%, 53.07%, 69.83% and 62.67%, respectively. The results above indicate a positive correlation between *BmToll9-2* expression and Toll pathway signaling gene expression in the fat body.

### 3.3. Silencing of BmToll9-2 Downregulated the Effector Genes

**Figure 3:**
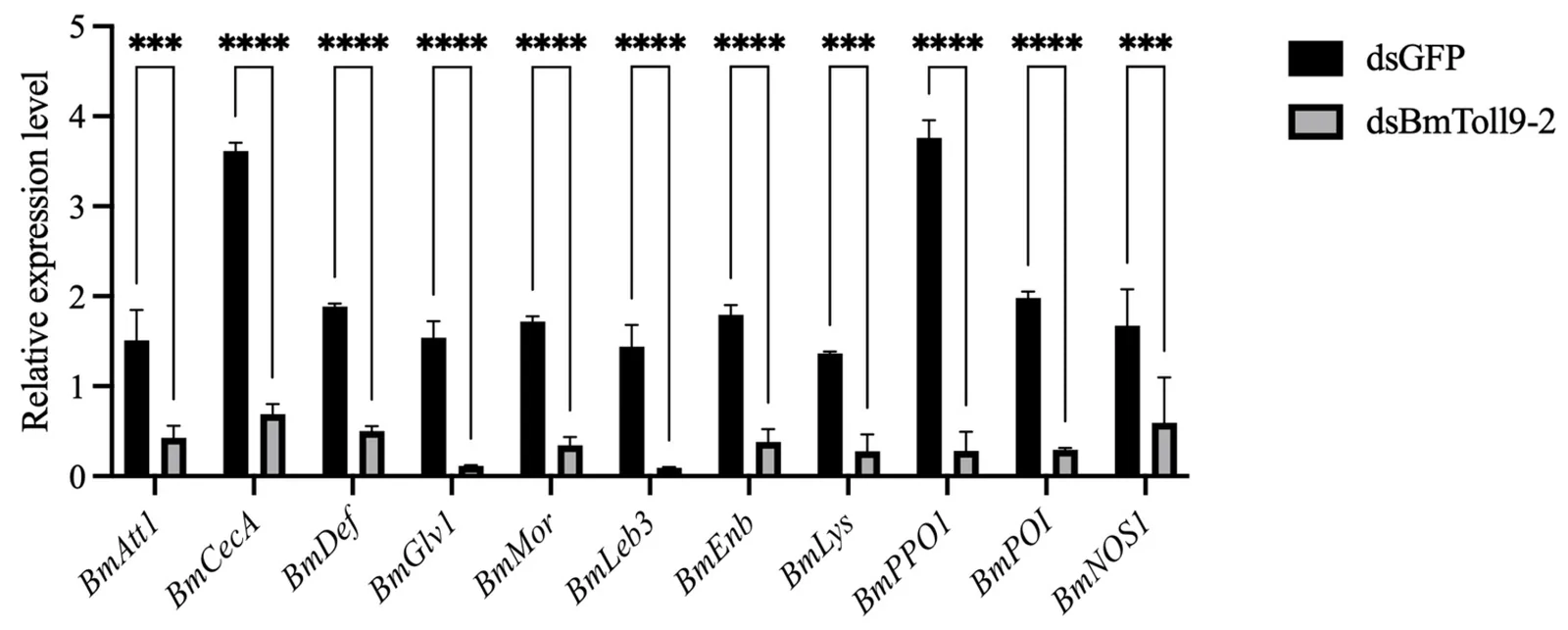
The relative expression of immune effector genes after the RNAi of *BmToll9-2* in the fat body. 5th-instar larvae were injected with ds*BmToll9-2*, and dsGFP served as a control. Data are represented as means ± standard deviations. Asterisks indicate significant differences from dsGFP injection groups (two-way ANOVA followed by Tukey’s HSD post-hoc test): *** *p* < 0.001; and **** *p* < 0.0001.

Similarly, to clarify the regulatory role of *BmToll9-2* on effector genes, the expression levels of 11 typical effector genes, including 7 AMPs, in the fat body were assessed post-RNAi (Figure 3).

The results indicated that compared to the dsGFP control group, the expression of all effector genes in the ds*BmToll9-2* treatment group was significantly downregulated. *BmAtt1*, *BmCecA*, *BmDef*, *BmGlv1*, *BmMor*, *BmLeb3*, and *BmEnb* (representing Attacin, Cecropin, Defensin, Gloverin, Moricin, Lebocin, and Enbocin gene families) were significantly downregulated by 71.85%, 80.91%, 73.47%, 92.53%, 79.94%, 93.40%, and 78.83%, respectively. Among other effector genes, *BmLys* (lysozyme) was significantly reduced by 79.85%. *BmPPO1* (prophenoloxidase) showed an extremely significantly 92.55% reduction. *BmPOI* (phenoloxidase inhibitor) was significantly decreased by 85.10%, and *BmNOS1* (nitric oxide synthase) by 64.48%. The results above demonstrate that the expression of the effector genes in the fat body is also positively correlated with the expression of *BmToll9-2*.

### 3.4. Bacterial Challenges Following RNAi Upregulated the Toll Pathway Signaling Genes

**Figure 4:**
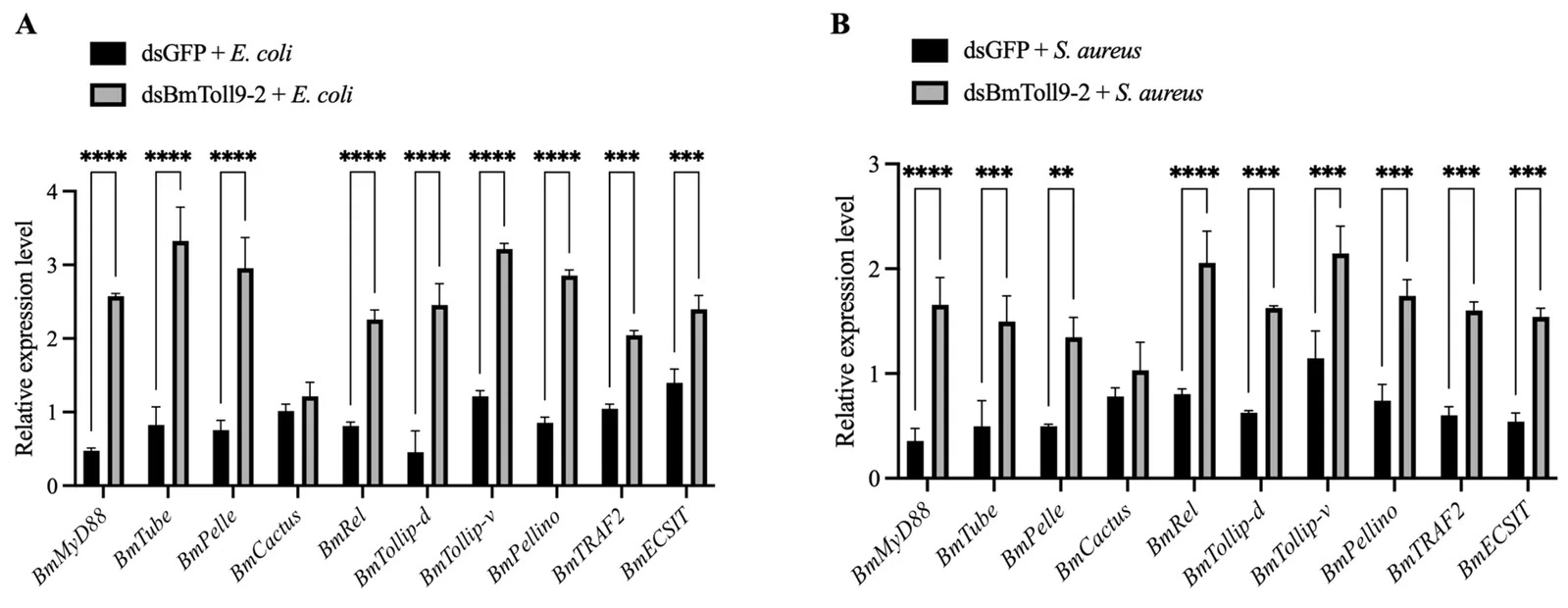
The relative expression of signaling genes in the Toll pathway following challenges with heat-inactivated bacteria after the RNAi of *BmToll9-2* in the fat body. 5th-instar larvae were injected with ds*BmToll9-2* or dsGFP. Then, the larvae were fed with heat-treated (**A**) *E. coli* or (**B**) *S. aureus*. Data are represented as means ± standard deviations. Asterisks indicate significant differences from dsGFP injection groups (two-way ANOVA followed by Tukey’s HSD post-hoc test): ** *p* < 0.01; *** *p* < 0.001; and **** *p* < 0.0001.

The results above showed that bacterial challenges post-RNAi upregulated the expression of *BmToll9-2*. Our previous investigation reported that bacterial challenges following RNAi upregulated the expression of the Toll pathway signaling genes in the midgut [23]. To further verify the triggering effect of bacterial challenges on the regulatory link between *BmToll9-2* and downstream components of the Toll pathway, signaling gene expression levels were detected in the fat body of silkworms after bacterial challenges post-RNAi (Figure 4).

Compared with the control group, feeding larvae with heat-inactivated *E. coli* post-RNAi upregulated the expression of most signaling genes (Figure 4A). Specifically, *BmMyD88*, *BmTube*, *BmPelle*, and *BmRel* were significantly induced by 5.42-, 4.03-, 3.91-, and 2.79-fold, respectively. *BmTollip-d* and *BmTollip-v* were significantly upregulated by 5.40- and 2.65-fold, respectively. And *BmPellino*, *BmTRAF2*, and *BmECSIT* were significantly induced by 3.34-, 1.96-, and 1.71-fold, respectively. In contrast, there was no significant difference in *BmCactus* expression between the two treatments.

Similarly, feeding larvae with heat-inactivated *S. aureus* post-RNAi also upregulated the expression of most signaling genes (Figure 4B). *BmMyD88*, *BmTube*, *BmPelle*, and *BmRel* were significantly induced by 4.66-, 3.02-, 2.72-, and 2.55-fold, respectively. *BmTollip-d* and *BmTollip-v* were significantly upregulated by 2.60- and 1.87-fold, respectively. And *BmPellino*, *BmTRAF2*, and *BmECSIT* were significantly induced by 2.35-, 2.67-, and 2.85-fold, respectively. No significant difference in *BmCactus* expression was observed between the two treatments.

The results revealed that bacterial challenges, both *E. coli* and *S. aureus*, upregulated the expression levels of most downstream signaling genes post-RNAi. Generally, *E. coli* exerted a stronger inductive effect compared to *S. aureus*.

### 3.5. Bacterial Challenges Following RNAi Upregulated the Effector Genes

Similarly, to further confirm the role of bacterial challenges in the link between *BmToll9-2* and the downstream effector genes of the Toll pathway, the effector genes were further investigated after bacterial challenges following the aforementioned RNAi.

**Figure 5:**
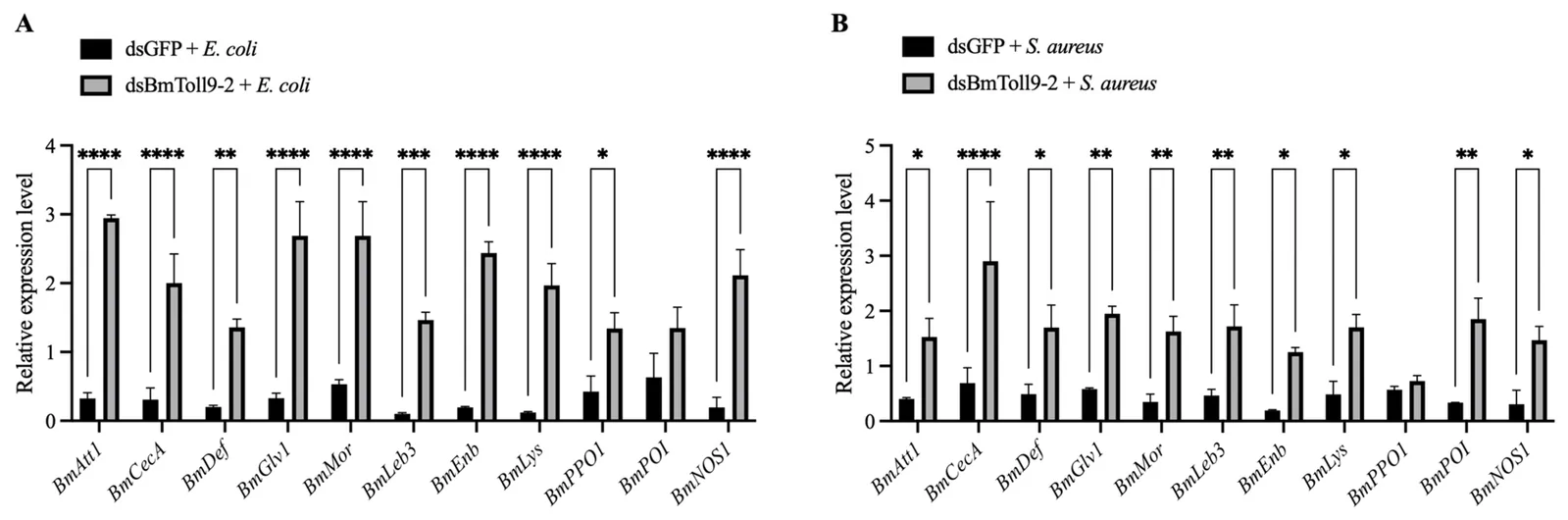
The relative expression of immune effector genes following challenges with heat-inactivated bacteria after the RNAi of *BmToll9-2* in the fat body. 5th-instar larvae were injected with ds*BmToll9-2* or dsGFP. Then, the larvae were fed with heat-treated (**A**) *E. coli* or (**B**) *S. aureus*. Data are represented as means ± standard deviations. Asterisks indicate significant differences from dsGFP injection groups (two-way ANOVA followed by Tukey’s HSD post-hoc test): * *p* < 0.05; ** *p* < 0.01; *** *p* < 0.001; and **** *p* < 0.0001.

After treating silkworms with heat-inactivated *E. coli* post-RNAi, the expression of most effector genes was induced to increase (Figure 5A). In the RNAi group, the AMP genes *BmAtt1*, *BmCecA*, *BmDef*, *BmGlv1*, *BmMor*, *BmLeb3*, and *BmEnb* were significantly upregulated by 9.06-, 6.48-, 6.73-, 8.21-, 5.06-, 14.31-, and 12.56-fold, respectively. Among other effector genes, except for *BmPOI* (with no statistically significant difference in relative expression), *BmLys*, *BmPPO1*, and *BmNOS1* were significantly induced by 16.21-fold, 3.16-fold, and 10.76-fold, respectively.

Similarly, feeding heat-inactivated *S. aureus* post-RNAi increased the expression of most effector genes (Figure 5B). In the RNAi group, the AMP genes *BmAtt1*, *BmCecA*, *BmDef*, *BmGlv1*, *BmMor*, *BmLeb3*, and *BmEnb* were significantly upregulated by 3.82-, 4.22-, 3.48-, 3.35-, 4.68-, 3.68-, and 6.46-fold, respectively. Among other effector genes, except for *BmPPO1* (with no statistically significant difference in relative expression), *BmLys*, *BmPOI*, and *BmNOS1* were significantly induced by 3.50-, 5.49-, and 4.82-fold, respectively.

The results indicated that bacterial challenges upregulated the expression levels of most downstream effector genes after *BmToll9-2* RNAi. However, the inductive effect of *E. coli* was stronger than that of *S. aureus*.

## 4. Discussion

Toll9 is a conserved membrane receptor in insects, primarily functioning in immunity [21]. The Gram-negative bacterium *E. coli* and its main cell wall component LPS, the Gram-positive bacterium *S. aureus* and its main cell wall component PGN could upregulated the expression of *BmToll9-2* [16]. However, the underlying mechanism in the fat body remained unclear. In this study, we investigated the role of *BmToll9-2* to bacterial challenges, and a regulator of the Toll signaling pathway and downstream effector genes in the *B. mori* fat body. Based on previous findings that silencing of *BmToll9-2* was effective in the fat body at 12 h [23], time points of 3 and 12 h were selected for analysis.

It is important to acknowledge that this study employed heat-inactivated bacteria rather than live pathogens. While immune stimulation induced by heat-inactivated bacteria is generally weaker and less sustained compared with live infection, heat-inactivated microbes retain intact cell wall components, including PGN and lipoteichoic acid in Gram-positive bacteria, as well as LPS from Gram-negative bacteria [32]. Heat-inactivation abolishes the replication capacity and pathogenicity of pathogens, preventing rapid mortality of experimental silkworms caused by severe infection [33]. Meanwhile, live bacteria secrete toxins and hemolytic factors, which would additionally trigger other damage-associated molecular pattern-mediated stress signaling pathways [34]. Therefore, the heat-inactivation approach allows us to isolate the specific effects of bacterial PAMPs on the Toll pathway without the confounding effects of active infection and pathogen-induced mortality. In addition, to maintain consistent experimental approaches with our previous midgut research, recapitulate natural bacterial infection routes of silkworms, and facilitate tissue-specific comparative analysis, we adopted the identical oral bacterial feeding paradigm in the present study.

### 4.1. BmToll9-2 Is a Potential Candidate for Responding to Bacteria Challenges in the Fat Body

Previously, we reported that *BmToll9-2* was found to be induced by bacterial challenges, particularly by the Gram-negative bacterium *E. coli*, both before and after RNAi-mediated silencing [16]. In the fat body, challenges with *E. coli* and *S. aureus* significantly upregulated the expression of *BmToll9-2* within 12 h, and the fold induction of *BmToll9-2* expression by the Gram-negative bacterium *E. coli* challenge was higher than that induced by the Gram-positive bacterium *S. aureus*. (Figure 1). Based on this observation, it is inferred that *BmToll9-2* in the fat body acts as a potential candidate for bacterial challenges. Although RNAi effectively silences the expression of *BmToll9-2* [23], the strong re-induction of the *BmToll9-2* upon bacterial stimulation poses a limitation: it precludes precise quantification of the actual knockdown efficiency during the stimulation phase.

*BmToll9-2* shares a close phylogenetic relationship with TLR4 [16], a well-characterized PRR for LPS detection in mammals [35]. Interestingly, recent evidence has demonstrated that its homolog, *BmToll9-1*, functions directly as a PRR capable of LPS recognition, analogous to TLR4 [12]. Given the structural and evolutionary conservation between these receptors, *BmToll9-2* represents a plausible candidate for LPS sensing. Consequently, it is reasonable that *BmToll9-2* may be transcriptionally responsive to *E. coli*, a Gram-negative bacterium whose outer membrane is abundant in LPS.

### 4.2. RNAi-Mediated Knockdown of BmToll9-2 Reveals Its Positive Role in the Toll Downstream Pathway Signaling in the Fat Body

In the Toll pathway, MyD88, Tube, Pelle, Cactus, and Rel are involved in signal transduction in the cytoplasm. Intracellular components such as Tollip, Pellino, TRAF2, and ECSIT are also factors involved in this process [9]. The successful RNAi of *BmToll9-2* resulted in significant downregulation of above Toll pathway signaling genes except *BmTRAF2* (Figure 2). The expression of *BmToll9-2* is elicited by bacterial challenges; specifically, this induction effect is pronounced in response to the Gram-negative bacterium *E. coli*, and it occurs irrespective of whether the RNAi of *BmToll9-2* is implemented beforehand or subsequently [16]. Accordingly, the identical samples subjected to bacterial challenges post-RNAi were examined; these samples exhibited marked upregulation of the majority of the Toll pathway signaling genes upon exposure to either *E. coli* or *S. aureus* (Figure 4). This suggests that *BmToll9-2* induction is accompanied by concurrent transcriptional upregulation of most downstream signaling genes. The relation of Toll genes to factors in the immune signaling pathway is extensively studied. In our previous study, the over-expression of *BmToll9-1* suppressed signaling genes in the IMD and JAK/STAT pathways after treatment with LPS [20]. *BmToll9-2* acted as a positive regulator of Toll pathway signaling genes in the midgut [21]. A parallel can be seen in the Chinese oak silkworm (*Antheraea pernyi*), where fungal-induced upregulation of the Toll gene triggered the induction of key pathway components, including MyD88, Cactus, and Rel [36].

Immune effector genes, including AMPs, exert direct interactions with infectious pathogens; these molecules accordingly mediate the neutralization of the targeted pathogens [15]. The successful RNAi of *BmToll9-2* resulted in significant reductions in most AMPs and other effector genes (Figure 3). Then, the identical samples subjected to bacterial challenges post RNAi treatment were examined; most AMPs and other effector genes upregulate exposure to either *E. coli* or *S. aureus* (Figure 5). Collectively, these findings indicate that *BmToll9-2* expression may correlates positively with immune effector production, suggesting its involvement in the transcriptional regulation of downstream immune responses. Previous studies have documented the regulation of AMP effectors by Toll receptors. We previously demonstrated that LPS treatment suppressed *BmToll9-1* and AMP transcription in *BmToll9-1*-overexpressing silkworm Bm5 cells [20]. More recently, we showed that *BmToll9-1* silencing reduced the expression of most effector genes in silkworm larvae [21]. Similarly, *BmToll9-2* acted as a positive regulator of Toll pathway signaling genes in the midgut [23]. Furthermore, silencing *BmToll9-1* impaired antibacterial activity [21], an effect recapitulated following *BmToll9-2* silencing [23]. However, the observed transcriptional recovery may also result from incomplete RNAi efficiency, or activation of parallel immune pathways that bypass *BmToll9-2* silencing. Protein-level validation such as Western blot and ELISA and functional assays are required to confirm whether the transcriptional changes translate into functional immune restoration.

These results suggest that, at the transcriptional level, *BmToll9-2* may positively regulate Toll signaling cascade in *B. mori*. However, further protein-level and functional studies are required to confirm this regulatory relationship.

### 4.3. BmToll9-2 Mediates Robust and Rapid Immune Signaling in the Silkworm Larval Fat Body

When comparing the transcriptional profiles observed in the present fat body data with those previously reported in the midgut under analogous experimental conditions [16,23], the fat body appeared to exhibit more pronounced transcriptional changes in both signaling and effector genes following *BmToll9-2* silencing and bacterial challenge. This observation hints at potentially distinct mechanistic roles of *BmToll9-2* between local gut immunity and systemic fat body immunity. This enhanced response is likely attributable to the fat body’s specialized cellular environment and molecular machinery, which are optimized for robust and rapid immune signaling. The differences in regulatory strength and speed between tissues likely reflect tissue-specific adaptations, with the fat body prioritizing immediate and strong immune responses to protect against systemic infections. In *D. melanogaster*, the fat body cells undergo rapid and dramatic changes in gene expression upon bacterial infection, mounting a robust and swift immune response [37]. Furthermore, the insect fat body rapidly initiate immune cascades upon infection, quickly upregulating immune-related genes to synthesize and secrete substantial quantities of AMPs for pathogen clearance [38]. These findings emphasize the critical role of the fat body in insect immunity and the importance of considering tissue-specific variations when studying immune pathway mechanisms.

It is also worth noting that several genes examined in this study, including *BmTube*, *BmPelle*, and *BmCactus*, are involved in developmental processes such as dorsal-ventral patterning and hematopoiesis, in addition to their roles in immunity. Therefore, the phenotypic and transcriptional changes observed following *BmToll9-2* silencing may partially reflect developmental perturbations rather than exclusively immunodeficient conditions [16]. The relationship between Toll pathway activity, developmental biology, and immune competence warrants further investigation.

## 5. Conclusions

This study focused on investigating the role of *BmToll9-2* in regulating the expression of downstream components of the Toll signaling pathway and the mechanisms underlying its crosstalk with effector genes in the larval fat body. Silencing *BmToll9-2* could lead to a decrease in the expression levels of both the Toll pathway signaling genes and effector genes. However, bacterial challenges following RNAi induced high expression of *BmToll9-2*, which simultaneously upregulated the expression of the Toll pathway signaling genes and effector genes. Collectively, these data support that *BmToll9-2* is a potential candidate in the fat body, and is positively involved in regulating the transcriptional activity of the Toll pathway signaling cascade and immune effector gene expression. Moreover, this study deepens our grasp of Lepidopteran innate immunity by providing transcriptional evidence for the regulatory role of *BmToll9-2* [39]. It also aids optimizing RNAi pest control, as the response of *BmToll9-2* to bacterial challenges offers targets for RNAi interventions [40,41].

## Figures and Tables

**Table 1:** List of primers used in this study.

Gene	Accession Number	Primer Sequence (5′–3′)
*BmToll9-2*	PP716770	F: GGTTACAAGCGAACGGTAGC
		R: CCAAATATCCGGACTGCTGC
*BmTIF4A*	DQ443290	F: TTCGTACTGGCTCTTCTCGT
		R: CAAAGTTGATAGCAATTCCCT
*BmTIF3s4*	DQ443238	F: ACTTCAAGTTCAGGGCAGAT
		R: TTAATTGTTTTGTGGAGGCT
*BmMyD88*	XM_028186400	F: AACGGTCACGACTCGAACTC
		R: TCTGCCCAGATTCTTCATCC
*BmTube*	XM_028173146	F: GGCAGAAAGTTATGGCTTGG
		R: ATCCTCAAATGCTCGCTGTT
*BmPelle*	XM_028182154	F: ACATCAAGCCGGCTAACATC
		R: ACCGTGAGACCTTCAGATGC
*BmCactus*	XM_028180230	F: ACAGTCGTGCGTACATTTGG
		R: CAGCCTCTCCCTATCGTCAA
*BmRel*	XM_028175224	F: TCGAATACATCCCGGACTTC
		R: TGGAAGGTCCTTTCTTGCTC
*BmTollip-d*	XM_021351983	F: GACGAGTCAGTCCCTCTTGC
		R: GTGGCTGGTGGAATTCGTAG
*BmTollip-v*	XM_028186930	F: TGCTACTTCTGACGGTGTGG
		R: AGGGCCACTTTGTGGTACTG
*BmPellino*	XM_028184930	F: AGAGTCGCTCAGCACAACAA
		R: CAATGTGGCTCCACACAGAT
*BmTRAF2*	XM_028172769	F: TCGCTCCTATGGGCATAACT
		R: CCGCATGTTGTGATTACTGG
*BmECSIT*	XM_028171307	F: ATGCCGCCTTAGCTAGAATG
		R: GCCTTTGGGCAGTACGTCTA
*BmAttacin1*	NM_001043541	F: CAGTGAACTCGGATGGAACC
(*BmAtt1*)		R: GGCGCTGAGTACGTTCTTGT
*BmCecropinA*	NM_001043997	F: CCGTCATAGGGCAAGCGAAA
(*BmCecA*)		R: AGCAATGACTGTGGTATGTCAA
*BmDefensin*	AB_367525	F: GTTAAGTGCGGCGTTGACTG
(*BmDef*)		R: TGACAGGGAAAGTGGAAGGG
*BmGloverin1*	AB_289654	F: GCTGGGATAGAAGCATCAGC
(*BmGlv1*)		R: ACATCAGGCCTTCTGTGACC
*BmMoricin*	AB_006915	F: TGTGGCAATGTCTCTGGTGT
(*BmMor*)		R: CTGGCGATATTGATGGCTCT
*BmLebocin3*	NM_001126260	F: CTCGATCCAAACCGAAGGTA
(*BmLeb3*)		R: CGGCTGGTCAAGTCCAGTAT
*BmEnbocin*	FJ373019	F: ACCTCGCACAACTAGTTCGG
(*BmEnb*)		R: CCAACAGAACAAACCCACTCG
*BmLysozyme*	NM_001043983	F: TAACGGCTCGAAGGACTACG
(*BmLys*)		R: GAGGTCGGAGCACTTAACGT
*Phenoloxidase inhibitor*	XR_001139981	F: GGATACGTGACTGGAAATGCA
(*BmPOI*)		R: GTCATAATCCACGGGTTTGTCC
*Prophenoloxidase 1*	AF_178462	F: AGTGGGAAGCCATTCTCCTT
(*BmPPO1*)		R: GCCAGGTTTCACTCCTTGAG
*Nitric oxide synthase1*	XM_012689821	F: TCATCACCACTAGCGCATCC
(*BmNOS1*)		R: CCTTGTCCGTTCTGTGTCCT
*T7-BmToll9-2*		F: TAATACGACTCACTATAGG TAGTATTCTCCCGGCTCTC
		R: TAATACGACTCACTATAGG GAAGGGTGCCTTGTGTAATC
*T7-GFP*		F: TAATACGACTCACTATAGG TACGGCGTGCAGTGCT
		R: TAATACGACTCACTATAGG TGATCGCGCTTCTCG

## Data Availability

The raw data supporting the conclusions of this article will be made available from the corresponding author upon reasonable request.
